# One-Step Method to Prepare PLLA Porous Microspheres in a High-Voltage Electrostatic Anti-Solvent Process

**DOI:** 10.3390/ma9050368

**Published:** 2016-05-13

**Authors:** Ying Wang, Li-Hui Zhu, Ai-Zheng Chen, Qiao Xu, Yu-Juan Hong, Shi-Bin Wang

**Affiliations:** 1 College of Chemical Engineering, Huaqiao University, Xiamen 361021, China; yingwang1019@sina.com (Y.W.); zhulihuihappy@163.com (L.-H.Z.); qiaoxu0218@sina.com (Q.X.); yujuan_hong@sina.com (Y.-J.H.); 2 Institute of Biomaterials and Tissue Engineering, Huaqiao University, Xiamen 361021, China; 3 Fujian Provincial Key Laboratory of Biochemical Technology, Huaqiao University, Xiamen 361021, China

**Keywords:** poly-l-lactide, porous microspheres, aerodynamic properties, pulmonary drug delivery, biosafety

## Abstract

A one-step method using a high-voltage electrostatic anti-solvent process was employed to fabricate poly-l-lactide (PLLA) porous microspheres (PMs). To address the simplification and control of the preparation process, a 2^4^ full factorial experiment was performed to optimize the operating process and analyze the effect of the factors on the morphology and aerodynamic properties of the PLLA PMs, and various characterization tests were also performed. The resulting PLLA PMs exhibited an even and porous morphology with a density less than 0.4 g/cm^3^, a geometric mean diameter (Dg) of 10–30 μm, an aerodynamic diameter (Da) of 1–5 μm, a fine particle fraction (FPF) of 56.3%, and a porosity of 76.2%, meeting the requirements for pulmonary drug delivery. The physicochemical characterizations reveal that no significant chemical change occurred in the PLLA during the process. An investigation of its *in vitro* cytotoxicity and pulmonary toxicity shows no obvious toxic response, indicating good biosafety. This study indicates that the one-step method using a high-voltage electrostatic anti-solvent process has great potential in developing an inhalable drug carrier for pulmonary drug delivery.

## 1. Introduction

Pulmonary drug delivery is a promising method of administration, transferring a drug by inhalation to treat lung disease or a systemic disease. Compared with oral and injected administration, pulmonary administration benefits from several favorable physiological features of the lungs [1,2]. For instance, they have a large surface area, a rich capillary network, and a thin layer of epithelial cells, perfect for absorbing and drug diffusion. Furthermore, the low biological activity of enzymes in the lung and the avoidance of the first-pass effect help to reduce the frequency of medication required and improve the efficacy of the drugs. Therefore, pulmonary drug delivery is an effective potential drug delivery route, suitable for the delivery of small proteins such as methotrexate [3], doxorubicin [4,5], and protein drugs such as insulin [6], exendin-4 [7,8], and lysozyme [9].

Porous microspheres (PMs) have been found to be effective tools for drug delivery because of their high deposition in the lung epithelium due to their low mass density (ρ < 0.4 g/cm^3^) and large geometric mean diameter (Dg, 10–30 μm), which facilitate the avoidance of phagocytic clearance [10,11]. Above all, the aerodynamic mean diameter (Da) has to be in the range of 1–5 μm, allowing the concentrated delivery of PMs to the deep lung [12,13].

Currently, strategies to fabricate microspheres include the emulsion method [14,15,16,17], the phase separation method [18,19], the collection medium [20], and the anti-solvent approach [21]. In general, especially for the double-emulsion method, the pores were formed by selecting different pore-forming agents, such as osmosis porogens [22,23], gas foaming porogens [24,25,26], and extractive porogens [27,28,29].

Many researchers have reported the obvious advantages of electrospraying in particle engineering, such as controllable particle shape, morphology, and particle size [30,31,32,33,34]. Our previous study [35] employed a process to fabricate large highly-porous microspheres (LHPMs) involving two steps, where the principle was a combination of an emulsion and a high-voltage electrostatic-based anti-solvent method [36,37]. It turned out that the resulting LHPMs had good dispersibility and a narrow size distribution. However, this process required preparing a stable water in oil (W/O) emulsion using Pluronic F-127 as an emulsifier at the first step, which was relatively complicated and might affect the biological safety of PMs due to the residue of emulsifier; further biological assessments of the products from this process have not been carried out.

In this study, using menthol as an extractable porogen, we attempted to use a one-step high-voltage electrostatic anti-solvent process without emulsion to prepare poly-l-lactide (PLLA) PMs that meet the requirements for pulmonary drug delivery. Instead of preparing a stable W/O emulsion and employing ammonium bicarbonate as a gas foaming porogen, as in our previous report [35], a simple homogeneous solution containing PLLA and menthol is directly introduced into the high-voltage electrostatic device to produce PLLA PMs in this one-step method. The physicochemical properties, *in vitro* cytotoxicity, and pulmonary toxicity of the PLLA PMs were also evaluated.

## 2. Results and Discussion

### 2.1. Optimization of the Process and Aerodynamic Properties of the PMs

**Figure 1:**
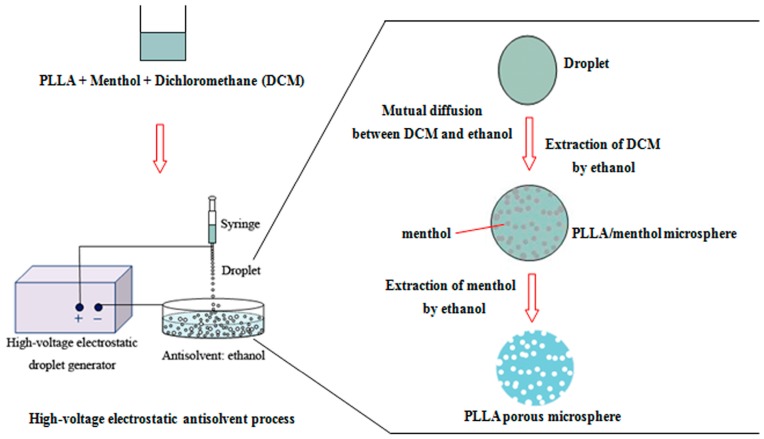
Schematic description of preparation of PLLA PMs.

In a high-voltage electrostatic anti-solvent process to prepare PLLA PMs, the droplet containing PLLA and menthol solution in DCM was sprayed into ethanol, the anti-solvent. As shown in Figure 1, the rapid mutual diffusion between DCM and ethanol generated a supersaturation of the PLLA solution, thus leading to the precipitation of PLLA as microspheres containing menthol microdroplets, which were subsequently extracted and removed by the large volume of ethanol, since menthol is soluble in ethanol, finally forming the PLLA PMs.

Table 1 shows the 24 full factorial experimental results on the sizes of the PMs, in which the span was defined as (D90−D10)/D50, a smaller span indicating a relatively narrow PM size distribution. The Dg and Da were about 20 μm and less than 4.7 μm, respectively, meeting the requirements for pulmonary drug delivery.

**Figure 2:**
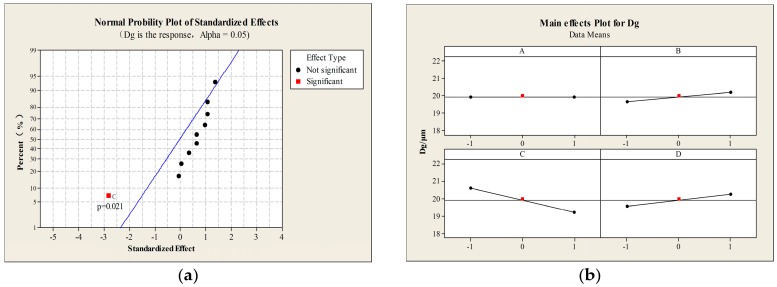

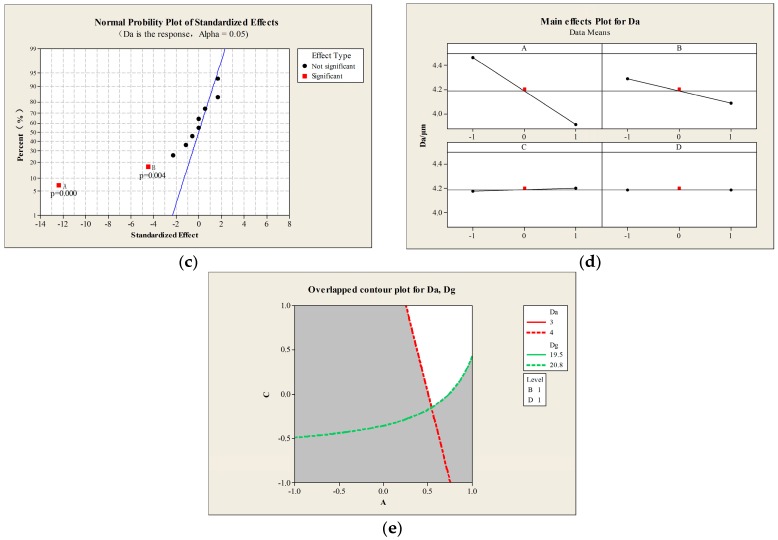
Minitab analysis: (**a**) Standardized effect of factors on Dg; (**b**) main effects plot for Dg; (**c**) standardized effect of factors on Da; (**d**) main effects plot for Da; (**e**) overlapped contour plot for both Da and Dg; (A) menthol: PLLA (w/w); (B) PLLA concentration (wt/v, %); (C) voltage (kV); and (D) push speed (mm/h).

Figure 2 shows the results of the statistical analysis by Minitab. The standardized effect plots are derived from the analysis of the fixed effects model, so the conclusions can only be applied to the factor levels considered in the analysis. The blue diagonal line shows a response that occurs when a factor changes from one level to another, and “not significant data points” means that the points exhibit a lack of influence. As shown in Figure 2a,b, the voltage is the only significant factor affecting the Dg (*P* < 0.05), and an increase in the voltage leads to a smaller Dg. Figure 2c shows that both the menthol:PLLA ratio and PLLA concentration had a significant effect on the Da with *P* < 0.01 and *P* < 0.05, respectively, which indicates that the role of the menthol: PLLA ratio is more significant than that of the PLLA concentration. This is also confirmed by the results of the main effects displayed in Figure 2d, in which the menthol: PLLA ratio (factor A) showed a bigger slope; the Da decreased with an increase in the menthol:PLLA ratio and the smaller the Da, the better for the deposition in the lung, which is favorable for targeted inhalation of the PMs to the lung. Figure 2e predicts that a higher voltage and menthol:PLLA ratio with the PLLA concentration and push speed maintained at a high level can obtain large PMs with a smaller Da. Based on the above results, we chose a PLLA concentration of 3%, menthol:PLLA ratio of 1:1, voltage of 10 kV, and push speed of 50 mm/h as the optimal conditions for the preparation of PMs for pulmonary drug delivery.

**Figure 3:**
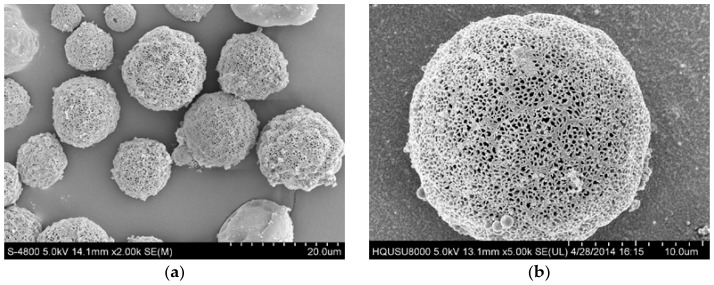

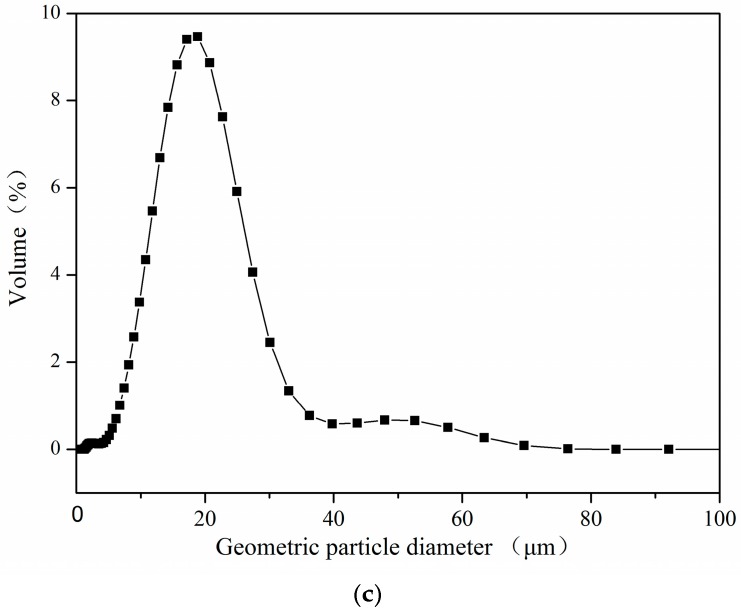
SEM image and particle size distribution of PLLA PMs. (**a**) ×2 k; (**b**) ×5 k; and (**c**) particle size distribution of PLLA PMs.

Figure 3 shows the morphology and size distribution of the PLLA PMs. The resulting PLLA PMs exhibit a large porous morphology, and the pores are dense with a narrow size distribution. The aerodynamic properties of the PLLA PMs are shown in Table 2. The mean Dg and Da of the PLLA PMs are 19.1 ± 0.4 μm and 4.2 ± 0.2 μm, respectively, which are suitable for a pulmonary drug delivery system. The FPF of the PLLA PMs is 56.3% ± 1.2%, and the density is 0.289 ± 0.004 g/cm^3^, which is less than 0.4 g/cm^3^, and therefore suitable for pulmonary administration. The mean pore size and porosity of the PLLA PMs are 0.2 μm and 76.2%, respectively, representing a larger specific surface area. These results demonstrate that menthol is an effective porogen for producing PLLA PMs with good properties for pulmonary drug delivery in the one-step high-voltage electrostatic anti-solvent process. This process avoids the preparation of a stable W/O emulsion and employment of ammonium bicarbonate as a gas-foaming porogen, as in the two-step method, while achieving a similar product of PLLA PMs [35], and this is favorable for its potential industrial application.

### 2.2. Physicochemical Properties of the PLLA PMs

**Figure 4:**
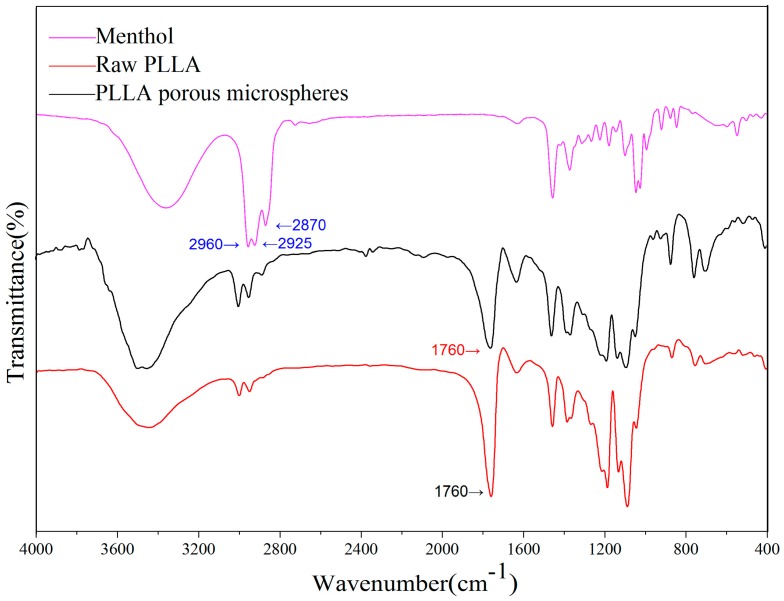
FTIR spectra of PLLA PMs.

The FTIR spectra of the PLLA PMs before and after the high-voltage electrostatic anti-solvent process are shown in Figure 4. As illustrated, the characteristic peak of PLLA (carbonyl, 1760 cm^−1^) [38] is retained in the spectrum of the PLLA PMs, indicating that the characteristic functional groups were not changed during the process. The characteristic peaks of menthol (methyl, methane, 2960 cm^−1^, 2870 cm^−1^, and 2925 cm^−1^) [39,40] are clearly observed in the spectrum of menthol, while these peaks are completely absent in the spectrum of the PLLA PMs, implying the complete removal of menthol from the PLLA PMs. This is also confirmed by the results of GC measurements (as shown in Figure S1 of the Supplementary Materials), which reveal that the peak of menthol has completely disappeared after the process. Similarly, as shown in Figure S2 of the Supplementary Materials, the level of DCM was too low to be detected (<41 ppm), which is far less than the maximum acceptable limit given in the Pharmacopeia of the People’s Republic of China (2010) (max. 600 ppm). This indicates that the one-step high-voltage electrostatic anti-solvent process is effective in removing the extractable porogen and the organic solvent residue, thus promoting the safety and suitability of the PLLA PMs for potential application in pulmonary drug delivery.

**Figure 5:**
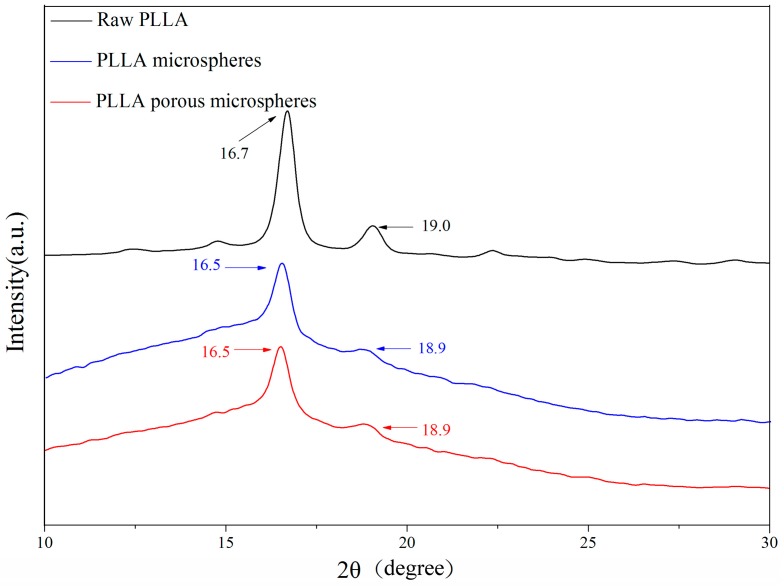
XRPD spectra of PLLA PMs.

**Figure 6:**
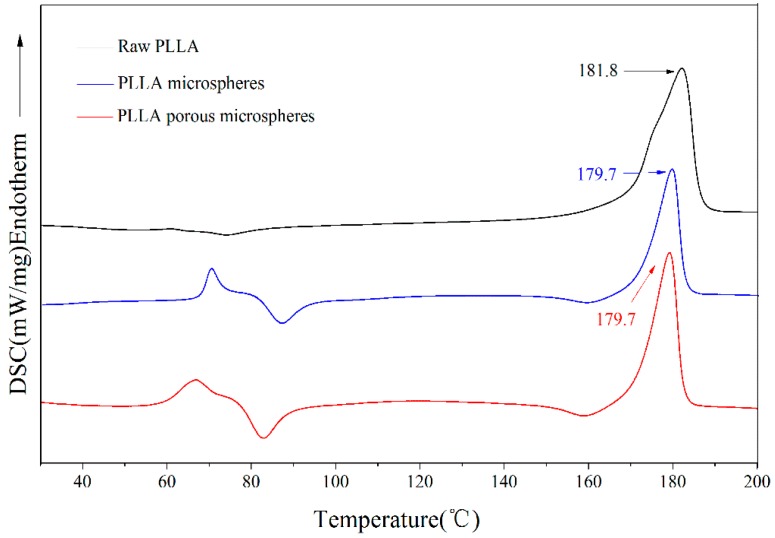
DSC curves of PLLA PMs.

Figure 5 shows the XRPD spectra of the PLLA PMs before and after processing. It is found that the characteristic diffraction peaks of the PLLA PMs (16.5° and 18.9°) are exactly the same as those of the solid PLLA microspheres formed without menthol as a porogen, while they are lower than those of the raw PLLA (16.7° and 19.0°). This indicates that the crystallinity of PLLA might be slightly reduced after re-crystallization, which reveals that a minor change in the physical state of the two crystal forms of α and α’ [41,42] occurred during the anti-solvent process, although the chemical component was not altered. This is also related to the results of DSC analysis, as shown in Figure 6.

As displayed, the characteristic melting peak of PLLA [43] slightly decreased from 181.8 °C to 179.7 °C after the high-voltage electrostatic anti-solvent process; the changes in the left part of the spectra with lower temperatures also indicate some changes in the glass transition temperature of PLLA. These both confirm the minor changes in the physical state of PLLA. The crystallinity of PLLA might be slightly reduced after re-crystallization during the anti-solvent process as the result of some slight hydrolysis.

### 2.3. In Vitro Cytotoxicity Study

**Figure 7:**
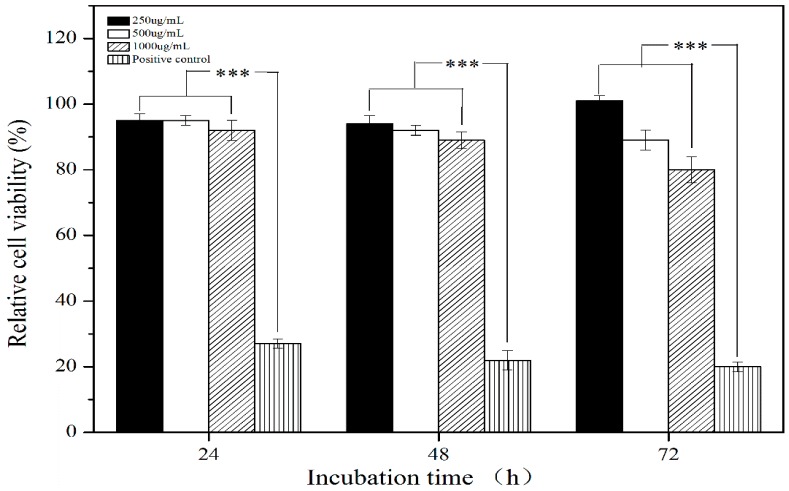
Relative cell viabilities of A549 cells cultured under different conditions. *** *P* < 0.01.

The cytotoxicity of PLLA PMs of different concentrations was tested by Alamar blue assay, and the results of relative cell viabilities in 24 h, 48 h, and 72 h are shown in Figure 7. It is demonstrated that the PLLA PMs of different concentrations have no cytotoxicity at different co-incubation times. Most of the A549 cells at each concentration grew well after 24 h, 48 h, and 72 h, with relative cell viabilities greater than 85%, indicating no significant cytotoxicity of the PLLA PMs, while the relative cell viabilities of the positive controls were only about 20%. The cell images (as shown in Figure S3 of the Supplementary Materials) also prove a corresponding phenomenon that the cells grew normally, spread well, and maintained a fusiform morphology within 72 h, while the cells of the positive controls became round and sparse, and tended to die. This result confirms the good biocompatibility of the PLLA PMs, and indicates that the one-step high-voltage electrostatic anti-solvent process maintains high biosafety of the raw PLLA for biomedical applications.

### 2.4. Pulmonary Toxicity Assessment

**Figure 8:**
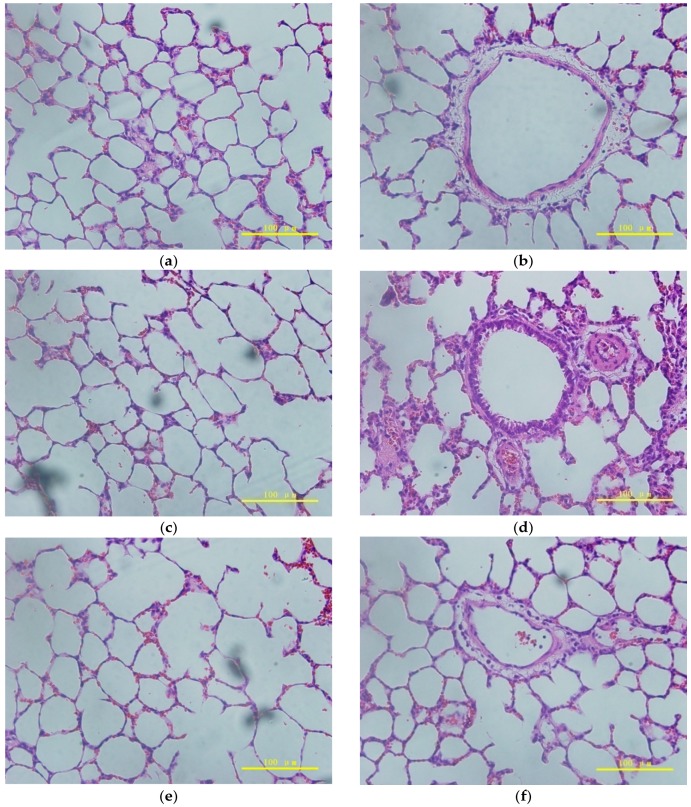

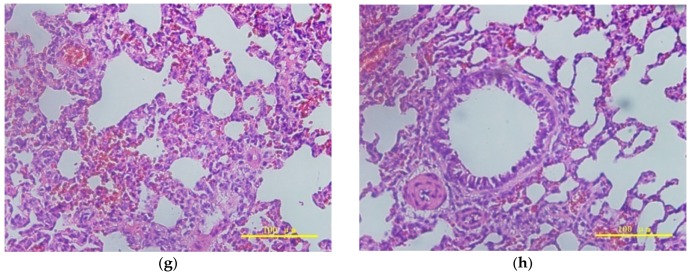
Histological examinations of lung tissues under different treatments: (**a**) pulmonary alveoli of the untreated; (**b**) trachea of the untreated; (**c**) pulmonary alveoli of air; (**d**) trachea of air; (**e**) pulmonary alveoli of PLLA PMs; (**f**) trachea of PLLA PMs; (**g**) pulmonary alveoli of LPS; and (**h**) trachea of LPS.

The pulmonary toxicity of the PLLA PMs with different treatments was tested by histological examination of the rats’ lung tissues, and the results are shown in Figure 8. As illustrated in Figure 8a,b, the alveoli of the untreated rats retained an elliptical morphology and tight connection of cells; the tracheas had an outspread shape with a thin wall. Figure 8c,d show that the alveoli of the air group had become a little bigger; the tracheas of the air group still showed a contractive morphology, the distance between cells had increased slightly, and a few blood and inflammatory cells were observed. Similarly, the alveoli of the PLLA PMs group in Figure 8e retained an elliptical morphology and the trachea in Figure 8f was contractive, indicating that the rats responded strongly after inhaling air or PMs. However, for the positive control, as illustrated in Figure 8g,h, the outline of the alveoli was unclear, with a large number of blood and inflammatory cells, the structure was thoroughly damaged, and the wall of the trachea had become thickened. According to these analyses, it can be concluded that the PLLA PMs themselves did not cause pulmonary toxicity to the lung tissues of the rats, while the blood and inflammatory cells which appeared in the alveoli and the minor changes in the trachea were caused by the normal physical defenses of the rats, which is definitely different from the chemical damages caused by LPS. This situation might be improved if the PLLA PMs were used in clinical trials, since the tracheas of rats are too small to undergo pulmonary administration without any damage.

**Figure 9:**
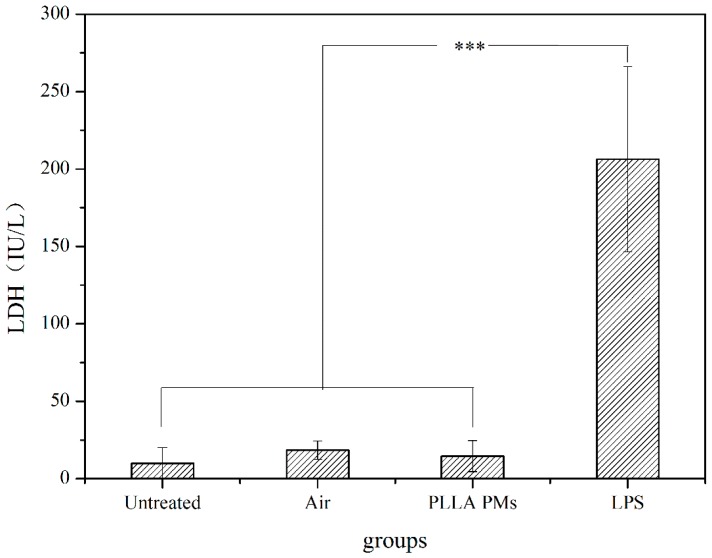
LDH levels of different treatments. *** *P* < 0.01.

Figure 9 shows the LDH levels of different treatments. LDH is a kind of endoenzyme and will escape from the cell when biological tissues are damaged, causing a high LDH level outside the cells. As illustrated in Figure 9, the untreated group had the lowest level of LDH, and the values of LDH increased slightly for the PLLA PMs group and the air group. However, the differences were not significant. These low levels of LDH were caused by the immune responses of the rats, and they were significantly lower than those of the LPS group. This result is consistent with the histological examinations of the lung tissues of the rats.

## 3. Materials and Methods

### 3.1. Materials

PLLA (Mw 50,000) was purchased from Jinan Daigang Biomaterial Co., Ltd. (Jinan, China). Menthol was purchased from Aladdin (Los Angeles, CA, USA). Dichloromethane (DCM, 99.8% purity) and ethanol (99.8% purity) were purchased from Sinopharm Chemical Reagent Co., Ltd. (Shanghai, China). Alamar blue was purchased from Invitrogen (Carlsbad, CA, USA). Human lung adenocarcinoma cell line A549 cells were obtained from Nanjing KeyGEN Biotech Co., Ltd. (Nanjing, China). SD rats were obtained from the Slac Laboratory Animal Co., Ltd. (Shanghai, China). A lactate dehydrogenase (LDH) kit was purchased from the Nanjing Jiancheng Bioengineering Institute (Nanjing, China). All other compounds were of analytical purity.

### 3.2. Methods

#### 3.2.1. High-Voltage Electrostatic Anti-Solvent Process

Figure 1 shows the schematic diagram of the preparation of PLLA PMs, which is a simplified version of that in our previous study [32]. Briefly, the homogeneous solution of PLLA and menthol at a certain concentration and ratio was placed into a 5-mL syringe and pumped into a container of ethanol through a blunt stainless steel 26 G nozzle (the inner diameter is 0.25 mm) by a microinfusion pump (AJ5805, Shanghai Anji Electronic Equipment Co., Ltd., Shanghai, China). The push speed is the flow rate of the polymer-solvent solution into the electrospraying nozzle. The high-voltage electrostatic field was generated by a high-voltage power supply (University of Shanghai for Science and Technology, Shanghai, China); the positive pole and negative pole of the high-voltage electrostatic droplet generator were attached to the needle and the take-up dish, respectively. When the pumping was finished, the resulting PLLA PMs were collected and dried in a vacuum for further characterizations.

#### 3.2.2. Full Factorial Experimental Design

In order to identify the optimal preparation conditions for the morphology and aerodynamic properties of PLLA PMs and key factors that affect these features, a 2^4^ full factorial experimental design was employed. As illustrated in Table 3, the factors (menthol: PLLA (w/w); PLLA concentration (wt/v, %); voltage (kV); and push speed (mm/h)) and their levels were selected based on the results of our previous experiments. Minitab software version 16 was used to design the experiments and analyze the influence and significance of the key factors on the Dg and Da.

#### 3.2.3. Surface Morphology Characterization

The surface morphology of the PLLA PMs was examined by scanning electron microscopy (S-4800 and SU8010 UHR FE-SEM, Hitachi, Tokyo, Japan). Dry samples were directly attached to specimen stubs with a conducting resin and then coated with a layer of gold.

#### 3.2.4. Aerodynamic Property Investigation

The Dg was measured by a laser particle size analyzer (LS13-320, Beckman Coulter, Kraemer Boulevard Brea, CA, USA). The Da was examined using a Mark II eight-stage Andersen Cascade Impactor (ACI20-810, Thermo Scientific, Waltham, MA, USA), which determined whether the PMs could reach the farthest parts of the lung. The samples (10 mg) were manually loaded into a capsule that was placed inside the dry powder inhaler device, with a hole ensuring the subsequent release of PMs. The inhaler device was then connected to the ACI by a stainless steel tube. Glass filters were placed on the ACI stages prior to the procedure to prevent particle bounce or re-entrainment [44]. The PMs were inhaled into the ACI at a flow rate of 28.3 L/min for 10 s and passed through each stage of the ACI. The amount of PMs deposited at each impaction stage was determined by measuring the difference in weight of the glass filters. The Da and fine particle fraction (FPF) were then calculated, the FPF defined as the amount of powder with an aerodynamic size <4.7 μm (PMs deposited at stage 3 and lower) according to the weight value. Furthermore, the tapped density of samples was also determined using a tapping apparatus (GH-01, Matsuhaku Electronic Co., Ltd., Xiamen, China). A sample of known weight was put into a 5-mL graduated cylinder and vibrated for 1000 taps up and down. The ρ was the ratio of the weight (g) and the volume (mL) after tapping until no further change in volume was detected. The pore sizes and porosities of the PMs were also measured using a high-pressure mercury intrusion porosimeter (Pore Master PM60-7-LP, Quantachrome, Boynton Beach, FL, USA).

#### 3.2.5. Physicochemical Properties of the PLLA PMs

In order to assess the influence of the process on the functional groups of the materials, Fourier transform infrared (FTIR) analysis was performed. Samples of raw PLLA and PMs were separately mixed with KBr and gently ground. The FTIR spectra were obtained using an FTIR analyzer (8400S, Shimadzu, Kyoto, Japan) over a wave number region of 4000–400 cm^−1^. X-ray powder diffraction (XRPD) was determined using an XRPD analyzer (D8 ADVANCE, BRUKER-AXS, Bremen, Germany) in the range of 5°–40°, with a step size of 2°/min, which was used to evaluate the change of the physical state of the materials after the process. Similarly, differential scanning calorimetry (DSC) was used in the range of 30–200 °C at a rate of 10 °C/min.

#### 3.2.6. Analysis of DCM Residue

The menthol residue was measured and analyzed by gas chromatography (GC, 19091N-113, Agilent, Santa Clara, CA, USA); the minimum detectable concentration is 1 μg/mL. The residue of the organic solvent DCM was measured and analyzed by GC (6890N, Agilent Technologies Inc., Santa Clara, CA, USA). Approximately 500 mg of the PLLA PMs was needed and the test was performed by the static head-space method according to the regulations of the Pharmacopeia of the People’s Republic of China (2010).

#### 3.2.7. *In Vitro* Cytotoxicity Investigation

The Alamar blue assay, one of the most common and effective methods for evaluation of the toxicity of biomaterials *in vitro*, was performed for the cytotoxicity test of the PLLA PMs. Human lung adenocarcinoma cell line A549 cells were cultured in Roswell Park Memorial Institute-1640 (RPMI-1640, Gibco Life Technologies, Waltham, MA, USA), supplemented with 15% (*v/v*) fetal bovine serum (FBS, Gibco Life Technologies, Waltham, MA, USA) and 1% penicillin/streptomycin at 37 °C and 5% CO_2_. Briefly, A549 cells were seeded at a density of 5 × 104 cells per well in a 96-well plate and subsequently co-incubated with leach liquors of the PLLA PMs at different concentrations (250, 500, and 1000 mg/mL) for 24, 48, and 72 h, respectively. The negative and positive controls were a blank medium and a medium containing phenol, respectively. After co-incubation, Alamar blue solution (10%, *v*/*v*) was added to each well for 4 h’ reaction. Finally, Alamar blue fluorescence examination was carried out at the excitation and emission wavelengths of 570 nm and 600 nm, respectively, and a microplate reader (Spectra Max M5, Molecular Devices, Silicon Valley, CA, USA) was used to measure the absorptions.

#### 3.2.8. Pulmonary Toxicity Assessment

In this part, all operative instruments had to be sterilized and disinfected for the treatment of rats. Pentobarbital solution was used at a certain dose to induce a short anesthesia adequate for pulmonary administration of the PLLA PMs to the rats. Dry PLLA PMs (3 mg) were directly administered into the lungs via the tracheas of the rats using a drug sprayer (DP-4-R, Penn-century, Philadelphia, PA, USA) with the help of a dedicated laryngoscope (LS-2-R, Penn-century, Philadelphia, PA, USA). The air group, the negative (untreated), and positive (LPS) controls were also assessed. Afterwards, the rats woke and regained normal breathing, diet, and physiological activity. After 4 h, the rats were executed, and one group of rats had their lungs and tracheas taken out. The specimens were treated with formalin, paraffin embedded, sectioned, hematoxylin and eosin (H & E) stained, and examined under a light microscope (BX51, OLYMPUS, Tokyo, Japan). Another group of rats was used to extract bronchoalveolar lavage fluid for LDH investigation.

## 4. Conclusions

PLLA PMs with a porous morphology, low density, and high porosity were successfully prepared by a one-step high-voltage electrostatic anti-solvent process, using menthol as an extractable porogen. It is proved that the Dg and Da of the PLLA PMs were suitable for pulmonary drug delivery. The chemical component of the materials remained unaltered, while minor changes in the physical state of PLLA occurred during the anti-solvent process. The complete removal of the porogen and organic solvent residue is favorable for the good biocompatibility of the resulting PLLA PMs, which were also proved to have no significant cytotoxicity or pulmonary toxicity. This work demonstrates that a one-step method using menthol as a porogen in a high-voltage electrostatic anti-solvent process is effective and promising for the production of polymeric PMs for pulmonary drug delivery.

## Figures and Tables

**Table 1:** Experiments and results of the Minitab design.

Run Order	Factors				Particle Sizes (μm)					
	A	B	C	D	D10	D50	D90	Dg	Span	Da
1	−1	1	−1	1	8.6	21.1	31.2	20.6	1.1	4.4
2	−1	−1	−1	−1	12.0	20.0	31.5	20.9	1.0	4.5
3	−1	−1	1	1	10.6	18.2	28.6	18.8	1.0	4.6
4	1	−1	−1	−1	10.0	19.5	31.5	20.2	1.0	3.9
5	1	1	1	1	11.7	19.6	29.8	20.1	0.9	3.9
6	−1	1	1	−1	9.9	17.7	27.8	18.2	1.0	4.3
7	−1	1	−1	−1	12.7	20.8	31.2	21.4	0.9	4.3
8	1	1	−1	1	13.2	21.3	30.4	21.5	0.8	3.9
9	−1	−1	−1	1	10.9	19.8	31.3	20.4	1.0	4.6
10	0	0	0	0	10.1	19.5	30.9	20.0	1.0	4.2
11	1	1	1	−1	11.6	19.9	29.6	20.2	0.9	3.8
12	1	−1	−1	1	9.1	19.9	32.8	20.9	1.2	4.0
13	−1	1	1	1	11.5	20.0	30.4	20.5	0.9	4.3
14	−1	−1	1	−1	9.0	17.6	29.0	18.4	1.1	4.7
15	1	−1	1	1	10.3	18.5	29.1	19.2	1.0	3.8
16	1	−1	1	−1	9.1	17.4	29.0	18.4	1.1	4.2
17	1	1	−1	−1	10.0	17.8	30.0	18.9	1.1	3.8

**Table 2:** Aerodynamic properties of the PLLA PMs.

Sample Name	Dg (μm)	Da (μm)	FPF (%)	Porosity (%)	Density (g/cm^3^)	Pore Size (μm)
PLLA PMs	19.1 ± 0.4	4.2 ± 0.2	56.3 ± 1.2	76.2	0.289 ± 0.004	0.2

**Table 3:** Experimental factors and levels.

Level	Code	A	B	C	D
		Menthol: PLLA (w/w)	PLLA Concentration (wt/v, %)	Voltage (kV)	Push Speed (mm/h)
High	+1	1:1	4.0%	10	50
Centre	0	3:4	3.5%	9	45
Low	−1	1:2	3.0%	8	40

## Supplementary Materials

The following are available online at www.mdpi.com/1996-1944/9/5/368/s1. Figure S1: GC of the PLLA PMs: (a) menthol; and (b) PLLA PMs; Figure S2: DCM residue of the PLLA PMs: (a) DCM, TR = 5.5; and (b) PLLA PMs, none of DCM residue; Figure S3: Optical images of A549 cells cultured for 48 h and 72 h: (a) 250 ug/mL—48 h; (b) 250 ug/mL—72 h; (c) 500 ug/mL—48 h; (d) 500 ug/mL—72 h; (e) 1000 ug/mL—48 h; (f) 1000 ug/mL—72 h; (g) the negative control—48 h; (h) the negative control—72 h; (i) the positive control—48 h; and (j) the positive control—72 h.
