# Supplementary material for: One-Step Method to Prepare PLLA Porous Microspheres in a High-Voltage Electrostatic Anti-Solvent Process
Source: Materials (Basel). 2016 May 13;9(5):368. doi: 10.3390/ma9050368 (PMC5503010; doi:10.3390/ma9050368)
Supplement: Supplementary file 1 [file materials-09-00368-s001.pdf]

Supplementary Materials: One-Step Method to Prepare PLLA Porous Microspheres in a High-Voltage Electrostatic Anti-Solvent Process

Ying Wang, Li-Hui Zhu, Ai-Zheng Chen, Qiao Xu, Yu-Juan Hong and Shi-Bin Wang

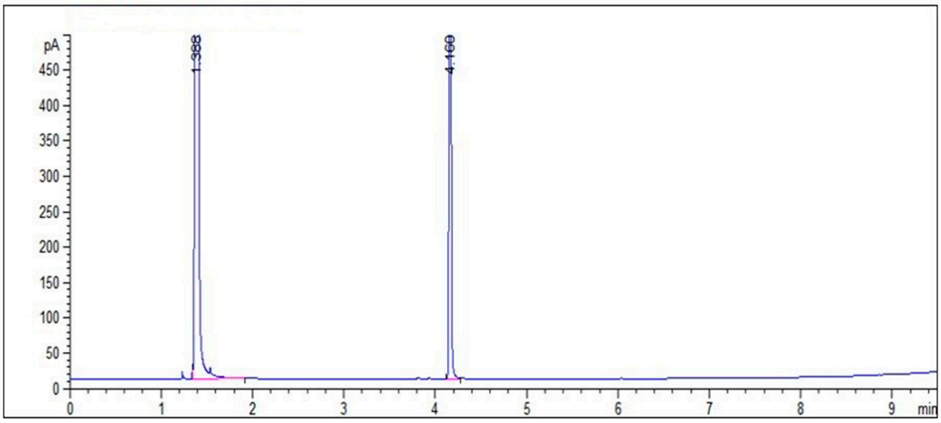

(**a**)

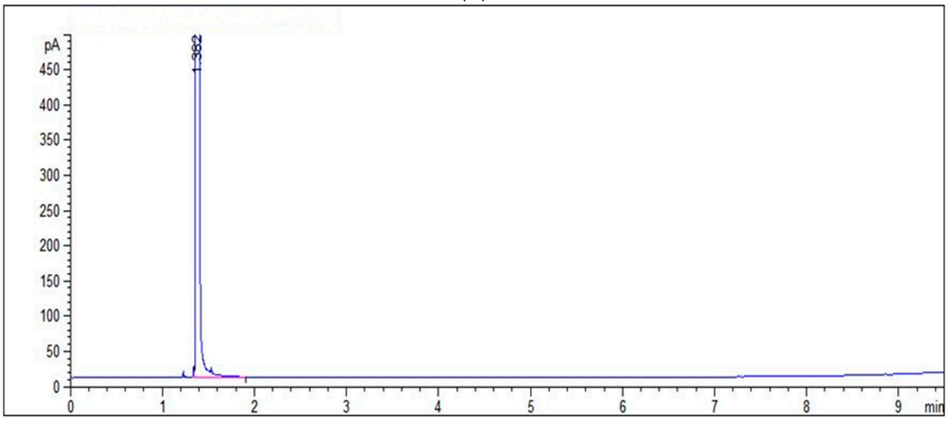

(**b**)

Figure S1. GC of the PLLA PMs: (**a**) menthol; and (**b**) PLLA PMs.

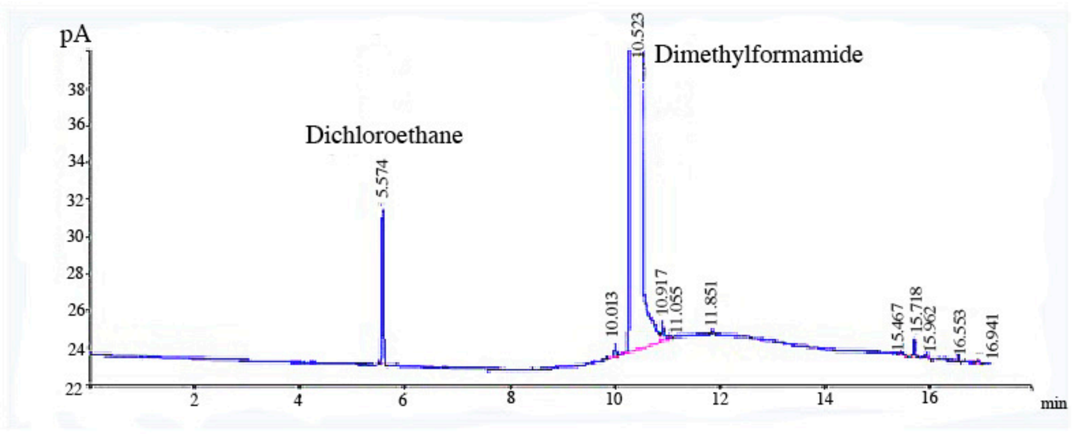

(a)

Figure S2. *Cont.*

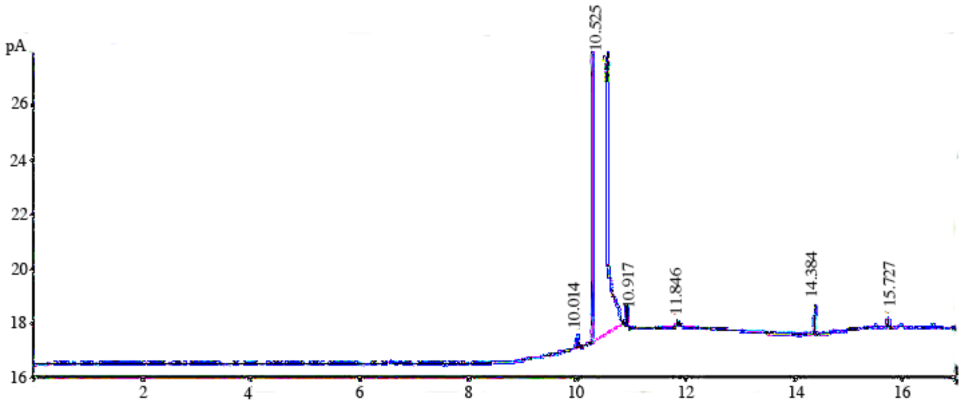

(**b**)

Figure S2. DCM residue of the PLLA PMs: (**a**) DCM, TR = 5.5; and (**b**) PLLA PMs with no of DCM residue.

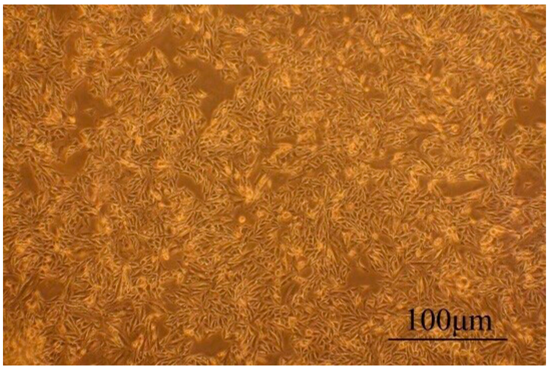

(**a**)

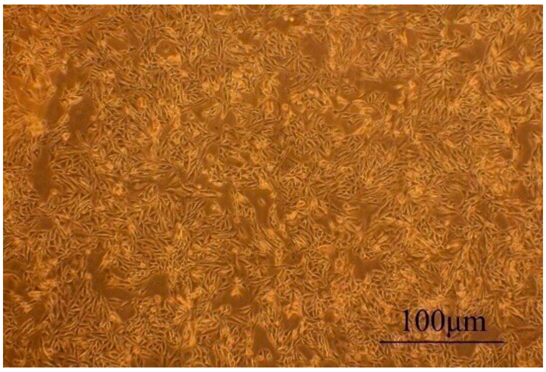

(**b**)

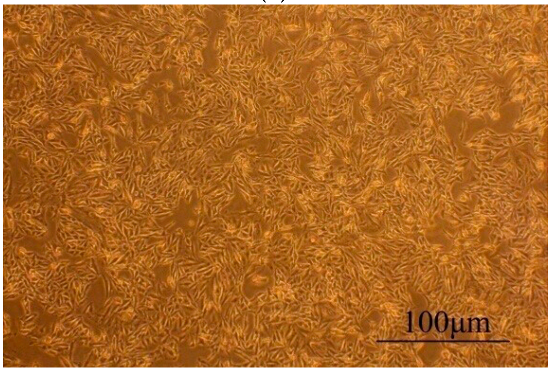

(**c**)

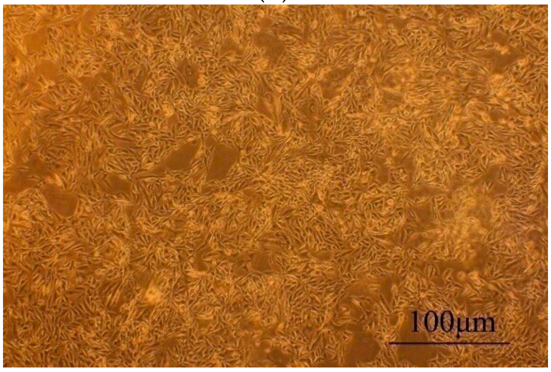

(**d**)

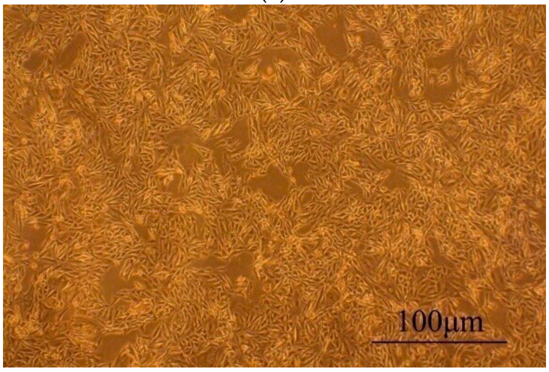

(**e**)

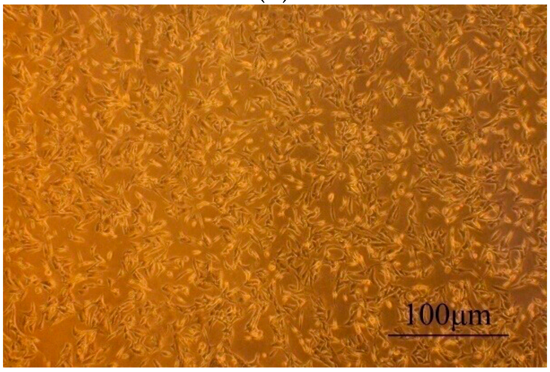

(**f**)

Figure S3. *Cont.*

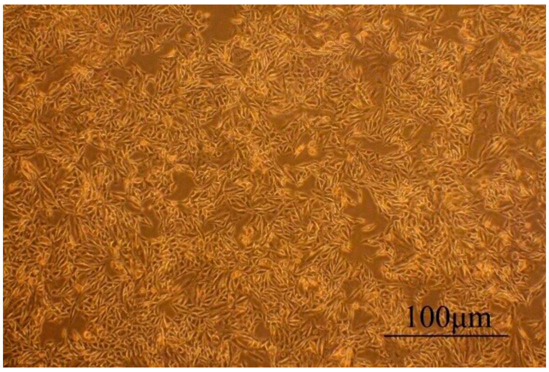

(**g**)

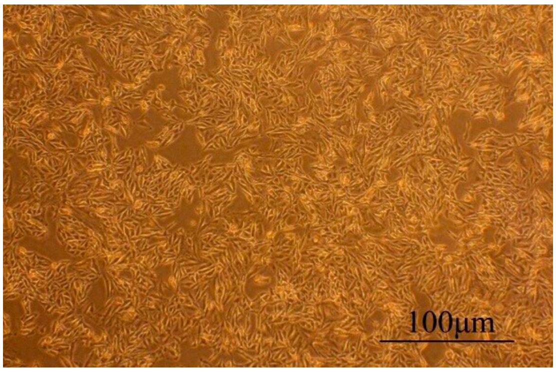

(**h**)

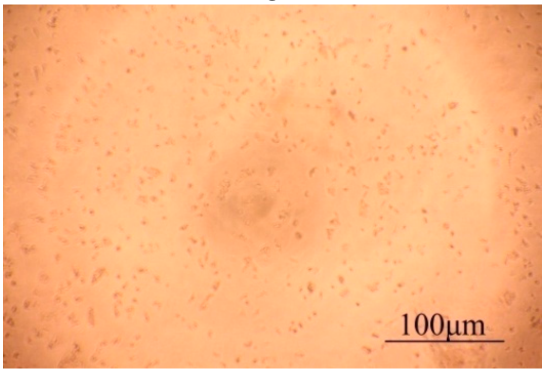

(**i**)

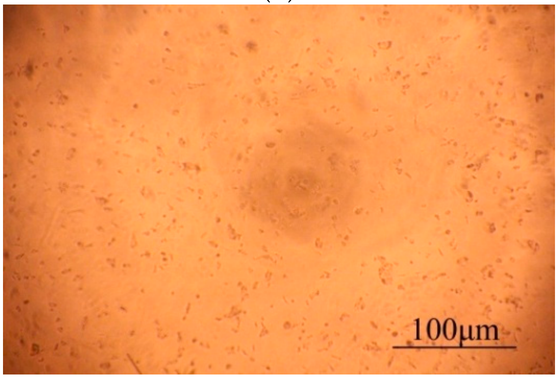

(**j**)

Figure S3. Optical images of A549 cells cultured for 48 h and 72 h: (**a**) 250 ug/mL-48 h; (**b**) 250 ug/mL-72 h; (**c**) 500 ug/mL-48 h; (**d**) 500 ug/mL-72 h; (**e**) 1000 ug/mL-48 h; (**f**) 1000 ug/mL-72 h; (**g**) the negative control-48 h; (**h**) the negative control-72 h; (**i**) the positive control-48 h; and (**j**) the positive control-72 h.
